# Selection signature analysis reveals *RDH5* performed key function in vision during sheep domestication process

**DOI:** 10.5194/aab-66-81-2023

**Published:** 2023-02-23

**Authors:** Ruixue Hu, Xunping Jiang, Huiguo Yang, Guiqiong Liu

**Affiliations:** 1 Laboratory of Small Ruminant Genetics, Breeding and Reproduction, College of Animal Science and Technology, Huazhong Agricultural University, Wuhan 430070, PR China; 2 Institute of Animal Husbandry, Xinjiang Academy of Animal Sciences, Urumqi 830000, PR China; 3 Key Laboratory of Agricultural Animal Genetics, Breeding and Reproduction of the Ministry of Education, Wuhan 430070, PR China

## Abstract

As one of the most successful domesticated animals in the Neolithic age, sheep gradually migrated all over the world with human activities. During the domestication process, remarkable changes have taken place in morphology, physiology, and behavior, resulting in different breeds with different characters via artificial and natural selection. However, the genetic background responsible for these phenotypic variations remains largely unclear. Here, we used whole genome resequencing technology to compare and analyze the genome differences between Asiatic mouflon wild sheep (*Ovis orientalis*) and Hu sheep (*Ovis aries*). A total of 755 genes were positively selected in the process of domestication and selection, and the genes related to sensory perception had directional evolution in the autosomal region, such as *OPRL1, LEF1, TAS1R3, ATF6, VSX2, MYO1A, RDH5*, and some novel genes. A missense mutation of c.T722C/p.M241T in exon 4 of *RDH5* existing in sheep were found, and the *T* allele was completely fixed in Hu sheep. In addition, the mutation with the *C* allele reduced the retinol dehydrogenase activity encoding by *RDH5*, which can impair retinoic acid metabolism and further influenced the visual cycle. Overall, our results showed significant enrichment for positively selected genes involved in sensory perception development during sheep domestication; *RDH5* and its variants may be related to the retinal degeneration in sheep. We infer that the wild sheep ancestors with weaker visual sensitivity were weeded out by humans, and the mutation was selective, swept by the dual pressures of natural and artificial selection.

## 1 Introduction

As an important livestock species domesticated by humans, sheep are one of the main resources of meat, wool, skin, and milk for humans since the Neolithic age. Due to the differences of habitat, production system, and breeding objectives, sheep have the greatest phenotypic diversity among different breeds. Previous studies have shown that compared with their wild ancestors, the decrease of brain volume and weight in domestication leads to more insensitive sensory organs and a more docile personality (Kruska, 1996, 1998).

Recently, the effects of domestication and subsequent selection on genomic variation in sheep have been studied, many quantitative trait loci (QTLs) and functional genes were found to be associated with phenotypic traits (Alberto et al., 2018; Naval-Sanchez et al., 2018; Zlobin et al., 2019). Selection signal and genome-wide association studies (GWAS) on wild ancestors were conducted, and they identified some selected regions and genes that may be affected by domestication (Li et al., 2020). To date, a series of nonsynonymous mutations and significant differences in allele frequencies among different breeds were also found, among which *PDGFD* may be an important gene affecting fat deposition in the sheep tail (Li et al., 2020; Zhu et al., 2021). In addition, based on comparative genomic analysis of sheep habitats in different environments, some candidate genes were detected to be associated with extreme environments, like the adaptability to high altitude hypoxia and water reservation in arid environments (Yang et al., 2016). Moreover, scholars also identified some selective sweeps related to important traits like horn size (Pan et al., 2018), wool color (Fariello et al., 2014), litter size (Yao et al., 2021), and variation of thoracic vertebrae (Li et al., 2019), by performing whole genome sequencing analysis. These findings suggested that during the domestication process, sheep adapted to their continuously changing environments under artificial and natural selection.

The ancestors of domesticated sheep mainly originated from the wild Asiatic mouflon sheep in southeastern Anatolia about 11 000 years ago (Demirci et al., 2013; Rezaei et al., 2010). Hu sheep are a unique yard-feeding local sheep breed in the Taihu Plain of China, which are famous for the following characteristics: no horn, short fat tail, docile disposition, early puberty, high fertility, and good lactation. We hypothesized that wild sheep must have a strong body size, skeletal system, and acute sensory perception under natural conditions due to the need to fight with natural enemies, the shortage of food in wild conditions, and other factors that led to a long time for wild sheep to achieve a very strong body. Some genes and mutations associated with growth and sensory perception remained during this domestication process. However, little is known about the genomic variation about Asiatic mouflon and Hu sheep to confirm our hypothesis. In the current study, to identify candidate genes related to sensory perception, we performed selection signal analysis between Hu sheep and Asiatic mouflon, and we found the potential variants and genes influencing sensory perception. Besides this, their effects on the structure and function were predicted and validated. This study provides a new insight into the adaptation of sheep to domestic environmental conditions.

## 2 Materials and methods

### 2.1 Samples collection and genome data resequencing analysis

For this study, sequencing data of 12 Asiatic mouflon and 59 Hu sheep are available from the National Center for Biotechnology Information (NCBI), and the data availability is presented at the end of this article. Then the FastQC (v0.11.5) software was used to detect the quality of the raw sequencing data. The information of the sequencing data and number of reads and bases in quality control is shown in the Supplement (Table S1). Meanwhile, the adapters, low quality bases (quality score <20 ), and reads shorter than 50 base pairs were filtered using Trimmomatic (v0.36) based on the quality control results. Besides this, all sequencing reads were aligned to the sheep reference sequence (Oar_v3.1) using Burrows–Wheeler Aligner (BWA) software. After mapping, we performed single-nucleotide polymorphism (SNP) calling based on Samtools (Li et al., 2009) and Genome Analysis Toolkit (GATK) v.3.7 (Mckenna et al., 2010).

### 2.2 Population structure analysis

To investigate genetic relationships between the Hu sheep population and the wild population, principal component analysis (PCA) was performed using the commands of PLINK (v.1.9) software (Purcell et al., 2007). Meanwhile, admixture (v.1.20) software was used to predict the ancestral groups and confounding situation of two sheep populations (Alexander and Lange, 2011). Besides this, linkage disequilibrium (LD) decay analysis was performed using PopLDdecay software (Zhang et al., 2019).

### 2.3 Selection signature detection

We used allele counts at variable sites to identify signals of selection in 150 kb windows sliding 50 kb using the average pooled heterozygosity ( $H_{P}$ ) and the average fixation index ( $F_{\mathrm{ST}}$ ) between wild mouflon sheep and domestic Hu sheep. The $H_{P}$ for populations was calculated using the following formula (Rubin et al., 2010):

1 $$H_{p}=\frac{2\times \sum n_{\mathrm{Maj}}\times \sum n_{\mathrm{Min}}}{{\left(\sum n_{\mathrm{Maj}}+\sum n_{\mathrm{Min}}\right)}^{2}},$$

where $n_{\mathrm{Maj}}$ represents the sum of the major allele frequencies of all SNP sites in the window and $n_{\mathrm{Min}}$ represents the sum of the minor allele frequencies. The $F_{\mathrm{ST}}$ for the population was calculated using the following formula (Weir and Cockerham, 1984):

2 $$F_{\mathrm{ST}}=\frac{\left(H_{T}-H_{S}\right)}{H_{T}},$$

where $H_{T}$ and $H_{S}$ represent heterozygosity in the total population and subgroup, respectively. According to a previous study, positive selected regions were located by extracting windows from the extreme tails of the Z -transformed $H_{P}$ and $F_{\mathrm{ST}}$ distributions by applying the cutoff that Z ( $H_{P}$ ) <-2.326 while Z ( $F_{\mathrm{ST}}$ ) >2.326 (Chong et al., 2022).

### 2.4 Gene ontology analysis

All variants were annotated by the Ensembl database (http://asia.ensembl.org/info/docs/tools/vep/index.html, last access: 16 December 2020) to identify genes located within positive selected regions. Furthermore, gene ontology enrichment analysis was performed using the PANTHER website (http://geneontology.org/, last access: 9 February 2021) (Ashburner et al., 2000; Mi et al., 2019). Cow orthologues of the sheep genes were converted and used as input. The significant enriched terms with the false discovery rate (FDR) value less than 0.05 were considered after adjustment by the Benjamini and Hochberg method (Benjamini and Hochberg, 1995).

### 2.5 Prediction of protein structure and physicochemical properties

According to the positive selection sites of *RDH5* candidate genes, the phylogenetic tree of different animals was conducted by MEGA X software (Kumar et al., 2018), and the three-dimensional (3D) structure of *RDH5* protein in sheep was predicted with the I-TASSER (http://zhanglab.ccmb.med.umich.edu/I-TASSER/, last access: 15 March 2021) online server. PyMOL v1.6.x was used to visualize the 3D structure and to locate the positive selected sites. Besides this, the physical and chemical properties of the *RDH5* protein were predicted by ProtParam (http://www.expasy.org/tools/protparam.html, last access: 16 March 2021) and ProtScale (https://web.expasy.org/protscale/, last access: 16 March 2021) (Garg et al., 2016).

### 2.6 Determination of retinol dehydrogenase activity

To detect the effect of positively selected sites on enzyme activity, the sequences of *RDH5* with wild (RDH5-T722) and mutation type (RDH5-722C) were synthesized based on the reference coding sequence in the Ensembl database (ENSOART00000012223.1). Then FLAG sequence was inserted before the stop codon of the two coding sequences separately. These two sequences were subcloned into pcDNA3.1 ( + ) vector by adding EcoRI and XhoI restriction sites at 5 ${}^{\prime }$ and 3 ${}^{\prime }$ ends, respectively. Next, DNA sequencing was used to detect the accuracy of the plasmid extracted by GoldHi EndoFree Plasmid Maxi Kit (Cowin Bio., CW2104M). Subsequently, HEK293T cells were cultured at 37 ${}^{\circ }$ C with 5 % CO ${}_{2}$ and 95 % humidity for 24 h in Dulbecco's Modified Eagle Medium (DMEM, high glucose 4.5 g L ${}^{-1}$ , 10 % fetal bovine serum, 1 % penicillin, and 1 % streptomycin). After that, the two plasmids mixed with Lipo8000™ Transfection Reagent (Beyotime, C0533) were transfected into HEK293T cells, respectively. Each transfection was repeated 3 times, and the cells were collected 48 h after transfection for protein extraction. Western blot was performed to verify whether the protein expression was successful. Finally, the enzyme concentration (ml003167) and activity (ml003319) were determined by following the instructions of the sheep *RDH5* ELISA kit (mlbio, Shanghai).

**Figure 1:**
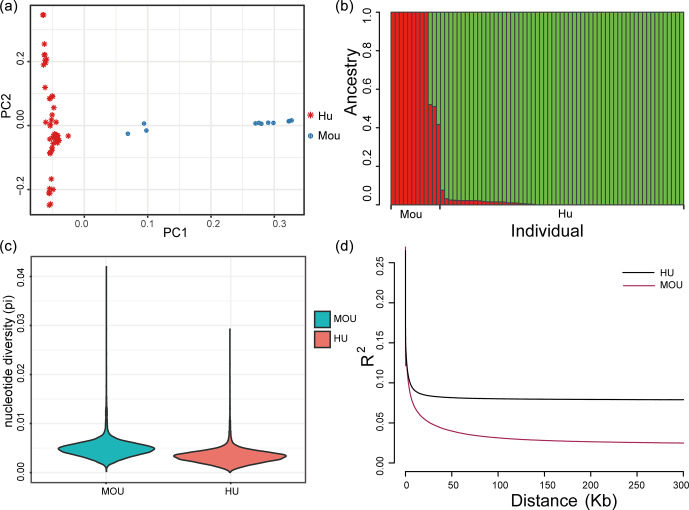
Population structure of the indigenous Hu sheep and Asiatic mouflon sheep populations. **(a)** Principal component analysis (PCA) plot of sheep populations; the first (PC1) and second (PC2) components were plotted, and the red and blue color represent Hu sheep and mouflon sheep, respectively. **(b)** Population genetic structure of ancestry compositions in a total of 71 sheep by ADMIXTURE, with the assumed number of ancestries being two. **(c)** The nucleotide diversity of the sheep population in this study. The blue block indicates the nucleotide diversity coefficient of the wild mouflon sheep population, and the red block indicates the nucleotide diversity coefficient of the domestic Hu sheep population. **(d)** Genome-wide linkage disequilibrium of sheep. The black line represents the wild sheep population, and the red line represents the domestic Hu sheep population; the LD of the domestic sheep population decayed faster than the wild sheep population.

## 3 Results

### 3.1 Genomic variants and population genetic structure

Population structure analysis was performed to estimate the genomic relationships of the two populations. The PCA result showed that the first component clearly divided Hu sheep and mouflon sheep into two sections (Fig. 1a). Besides this, to validate the PCA results, we further analyzed population structure using ADMIXTURE, and the optimal modeling choice occurred when the assumed number of ancestries is two (Fig. 1b). There were differences in pedigree purity within the mouflon sheep population and among the Hu sheep population. The x axis indicated each individual, and the y axis indicated the proportion of specific ancestral lineages. A higher proportion of a certain color indicated a higher purity of the blood relationship between the individual and a certain ancestor. Moreover, the average nucleotide diversity (pi) of mouflon sheep was 0.0049, while for Hu sheep it was 0.0033, and the results confirmed the average pi of wild mouflon sheep is higher than domestic Hu sheep (Fig. 1c). Furthermore, the result of LD decay analysis was nearly consistent with the nucleotide diversity of the sheep population, in which the lowest genetic diversity was found in the Hu sheep breed (Fig. 1d). The clear genetic divergence between the wild mouflon sheep and the domestic Hu sheep indicated that these individuals were adequate to perform further selection signature analysis.

**Figure 2:**
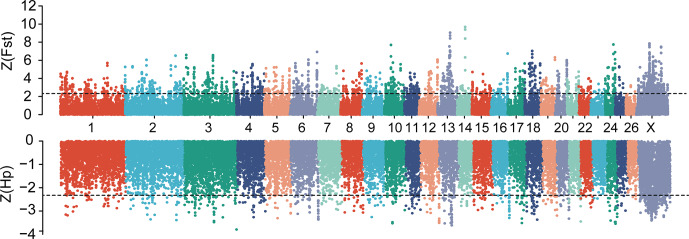
Manhattan plot of genome-wide selective sweep signals ( Z ( $F_{\mathrm{st}}$ ) and Z ( $H_{p}$ ) ${}_{\mathrm{HU}}$ ) in two sheep breeds. For each metric, a 150 kb sliding window with a step size of 50 kb was applied. The positive selected regions were extracted at the extreme ends of the distribution by applying Z ( $F_{\mathrm{st}}$ ) >2.326 and Z ( $H_{p}$ ) <-2.326 cutoffs.

### 3.2 Genome scans for selection signature and functional enrichment analysis

**Figure 3:**
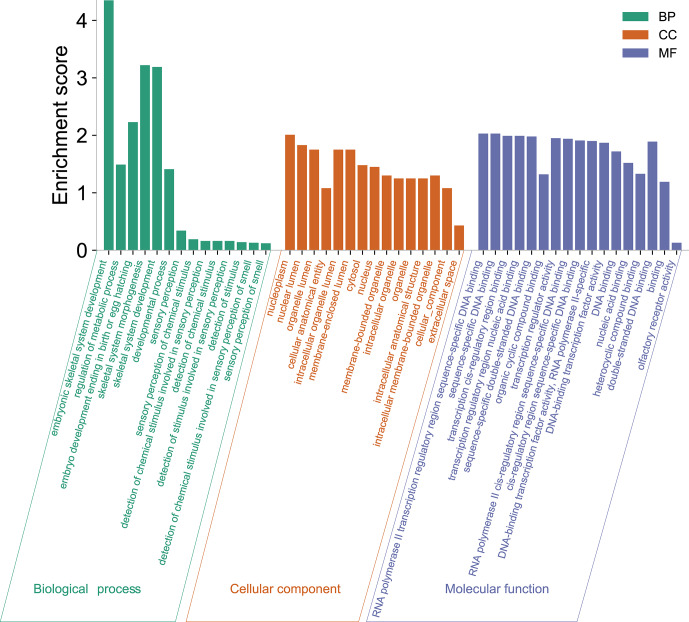
Functional classification of 755 positive selected genes across wild Asiatic mouflon sheep and domestic Hu sheep population. Green, orange, and purple represent the biological process, cellular component, and molecular function, respectively.

In this study, to detect the selection signal between mouflon and Hu sheep, we performed a selective sweep analysis for regions with pooled heterozygosity ( $H_{p}$ ) of Hu sheep based on Z ( $H_{p}$ ) ${}_{\mathrm{HU}}$ <-2.326 and increased genetic distance to mouflon sheep based on Z ( $F_{\mathrm{st}}$ ) >2.326 of a window of 150 k and a step of 50 k over the whole genome, respectively (Fig. 2). We identified a total of 589 regions with elevated $F_{\mathrm{ST}}$ and 445 regions with $H_{p}$ of Hu sheep. Then 288 overlapped regions were considered as positive selected regions by the intersection of the two approaches. Finally, 755 positive selected genes were identified via using the Ensembl gene annotations within positive selected regions (Table S2). Among these 755 genes, only 128 genes had missense mutations (Table S3). Moreover, gene ontology enrichment analysis of 755 candidate genes was performed by the Gene ontology program; the results are shown in Fig. 3 and Table S4. In the biological process, we found most genes mainly enriched in biosynthetic and metabolic process terms, skeletal system development, and other life activities (Fig. 3). Interestingly, we also found 10 genes involved in sensory perception terms including taste 1 receptor member 3 (*TAS1R3*), lymphoid enhancer binding factor 1 (*LEF1*), retinol dehydrogenase 5 (*RDH5*), opioid-related nociceptin receptor 1 (*OPRL1*), activating transcription factor 6 (*ATF6*), visual system homeobox 2 (*VSX2*), myosin 1A (*MYO1A*), and three novel genes which have no gene name currently (Table S5). In these 10 genes enriched in sensory terms, only *RDH5* and *ATF6* belonging to this term had missense mutations.

### 3.3 The *T/C* polymorphism at the 722th site in *RDH5* changes enzyme activity

**Figure 4:**
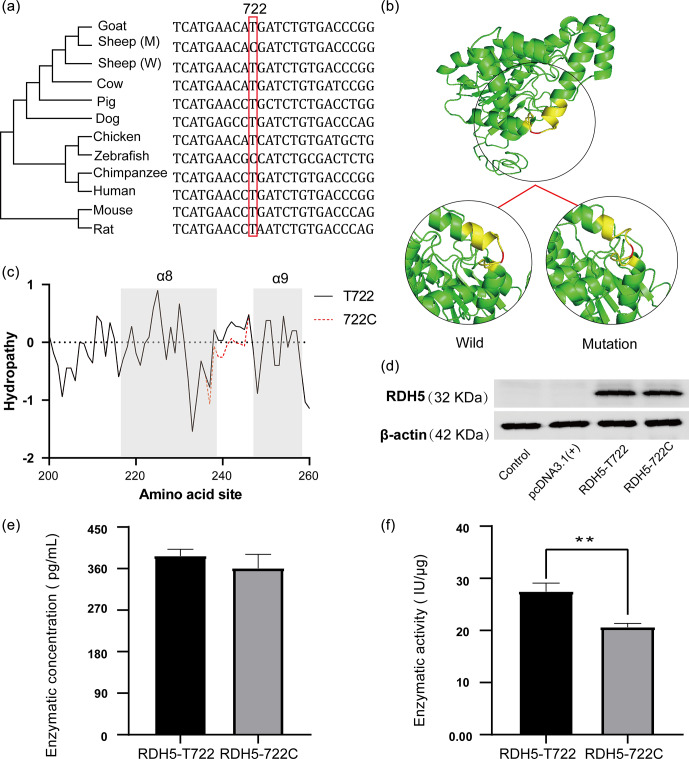
The information of the *RDH5* evolutionary tree and changes of mutation in protein structure and hydrophilicity. **(a)** Evolutionary analysis of the c.722A > G amino acid variant. The phylogenetic tree derived from the multiple alignment was constructed; **(b)** *RDH5* c.722T > C mutation changes the structure of sheep *RDH5* protein. Compared with c.722 C-type *RDH5* protein, the corresponding α helix of c.722 T-type *RDH5* protein is extended, and the spatial conformation of the protein changes. **(c)** *RDH5* c.722 T > C mutation significantly enhances the hydrophilicity of amino acid residues 234–242; **(d)** western blot results of the *RDH5* protein levels; **(e)** the enzyme concentration of *RDH5*-wild and *RDH5*-mutation; and **(f)** the enzyme activity of the *RDH5*-wild and *RDH5*-mutation ( ${}^{**}$ represents p<0.01 ).

After rigorous screening, a positively selected SNP locus (*RDH5* c.722 T > C) was found to be functionally verified. The basic situation is shown in Fig. 4. The *RDH5* c.722 T > C locus is located in the exon 4 of *RDH5*, at the 722 ${}_{\mathrm{th}}$ nucleotide of the coding sequence (CDS). This is a missense mutation site, which causes the 241st amino acid of the *RDH5* protein to be converted from methionine to threonine. In domestic Hu sheep and wild Asiatic mouflon populations, the frequency of allele *T* in domestic sheep is 1 and allele *C* in wild sheep is 0.292, respectively (Fig. S1).

In order to study the selection of *RDH5* c.722 T > C sites among species, a phylogenetic tree was constructed based on the *RDH5* coding sequence of different animals, and the detailed source of the sequence is shown in Table S6. It can be found that c.722 T > C sites are a new mutation and the artiodactyl animals are closely tied together (Fig. 4a). Besides, the prediction of 3D protein structure showed that this variation affects the spatial position of adjacent alpha helices (Fig. 4b). In addition, the mutation site strengthened the hydropathicity of the partial α 8 helix (Fig. 4c).

In order to validate the influence of the missense mutation to the expression of the *RDH5* protein, two eukaryotic expression vectors of T722C were constructed and transfected into HEK293T cells (Fig. S2), and western blot detection verified that both plasmids were successfully expressed *RDH5* protein (Fig. 4d). Besides this, we determined the enzyme content and enzyme activity in the protein; the retinol dehydrogenase activity of the *RDH5*-wild is 27.45 ± 1.34 IU µ g ${}^{-1}$ and in the *RDH5*-mutation it is 20.62 ± 0.60 IU µ g ${}^{-1}$ , which confirmed a significant difference ( p<0.01 ) (Fig. 4e, f).

## 4 Discussion

An analysis of the population genetic structure provides important clues to elucidate the genetic relationships and identify mutations responsible for improved traits between populations (Slatkin et al., 1987). The results of PCA and population structure revealed a clear separation between wild Asiatic mouflon and domestic Hu sheep. Meanwhile, there was some gene flow between the two populations (Fig. 1a and b). A previous study verified that introgression between wild and domestic sheep can improve the ability to resist pneumonia (Cao et al., 2021). This suggested genetic introgression of wild Asiatic mouflon sheep into domestic Hu sheep through human migration and other activities.

Moreover, we explored the variome and selective sweeps via comparing the whole genome of wild and domesticated sheep; the nucleotide diversity of Hu sheep was lower than that of Asiatic mouflon, which was consistent with the results of LD decay (Fig. 1c and d). A previous study confirmed that the level of genetic diversity was positively correlated with the survival and evolutionary potential of the species or populations (Li et al., 2017). Our results were also consistent with the fact that wild sheep with high levels of genetic diversity are better at adapting to a complex natural environment. Vast amounts of genomic variation have been lost under the dual pressures of natural selection and domestication.

Besides this, we found some known functions for these positive selected genes, such as *BMPR1B* and *B4GALNT2* for sheep litter size (R. Talebi et al., 2018), and *RXFP2* for horn type, which was consistent with a previous study (Pan et al., 2018). Interestingly, we also found genes enriched in the sensory perception pathway. Sensory perception, including vision, audition, olfaction, and tactus, plays critical functions in individual survival and behaviors like foraging, evading predators, mate recognition, and so on (MacDonald et al., 2006). For example, it has been confirmed that the visual sense is vital for the survival of birds (Fernández-Juricic et al., 2012). Studies in red junglefowl and village chickens revealed that the positive selection on vision-related genes like *VIT* drives the weaker visual acuity during their domestic process, which was verified by the knockdown of *VIT* in zebrafish (Wang et al., 2016). Meanwhile, other sensory abilities including smell, taste, and hearing also played an outsize role in domestic animals (Martinac et al., 2022). As one of the most valuable modes of sensory perception, olfactory sensation provides the basis for the extraordinary sensitivity needed to distinguish between environment and gender (Assanasen et al., 2014). Many species can locate food, avoid danger, identify spouses, and recognize territory by sensing the chemical molecules in the habitat environment (Izquierdo et al., 2018). Meanwhile, the taste system is also crucial in analyzing the food ingredients and deciding whether to eat it or not. Taste perception is crucial, as animals not only need energy for survival but also need to avoid toxic chemical compounds (Dutta et al., 2020). Unlike pigs, chickens, and other livestock feeding on formula feed, for herbivores such as sheep, smell plays a very important role in the identification and selection of foraged species. In the reproduction process, ewes isolated from rams for a long time can promote ewes to estrus in advance when smelling the odor of rams (Niimura et al., 2014; Gelez et al., 2004). Besides this, ewes can specifically recognize their lamb's voices and breastfeed their lambs after childbirth (Sebe et al., 2007). Therefore, these typical characteristics can explain the results of the current study of the variation of sheep in the domestic process.

In the previous discussion, we mentioned that sensory perception is related to many behaviors, and the evolution of these behaviors will inevitably promote the genes including *TAS1R3, VSX2, LEF1, OPRL1, ATF6, MYO1A, RDH5*, and so on, related to sensory perception, showing signs of molecular adaptation in the process of domestication.

### TAS1R3

As a main receptor for sweet taste, *TAS1R3* is a member of G protein-coupled receptors family C, which can medicate sweetness preference. A study in 30 mouse strains identified that the polymorphism in the *TAS1R3* region have a special fondness for saccharin (Reed et al., 2004). Besides, *TAS1R3* deletion impaired glucose and insulin tolerance in mice (Murovets et al., 2015). Recent studies also revealed that the allelic variation of the *TAS1R3* gene influences the preference for sweet taste and reduces plasma insulin in F1 mouse hybrids (Murovets et al., 2020). Two SNPs of rs307355 and rs35744813 located at the upstream of the coding region of *TAS1R3* are associated with human gustatory sensitivity to sucrose (Fushan et al., 2009). Further study is required to verify the function of this gene.

### VSX2

As a transcription factor, *VSX2* plays an important role in the development of retinal progenitor cells which eventually causes all nerve tissues in the retina. Mutations in *VSX2* can lead to autosomal recessive microphthalmia (Reis et al., 2011). Besides this, by targeting CRISPR/Cas9 to knock out either the coding sequences or enhancers of *PRDM1* and *VSX2*, both the number of formed photoreceptors and bipolar cells were affected. This evidence suggests that *VSX2* is indispensable for maintaining bipolar fate (Goodson et al., 2020). This evidence suggests that *VSX2* missense mutations may cause loss of gene function and lead to the mutant death.

### LEF1

As a transcription factor belonging to the Tcf/Lef family, *LEF1* performed an indispensable role in the formation of whiskers in rodents by acting in the upstream of Prdm1 (Manti et al., 2022). RNA-seq analysis of chickens between gustatory epithelium and ongustatory mesenchyme/connective tissue showed that *LEF1* was a differentially expressed gene, suggesting *LEF1* is essential for the development of taste organs (Cui et al., 2017). *LEF1* can regulate osteogenic differentiation and promote tooth organogenesis (Luo et al., 2019; Kratochwil et al., 2002). In a high-altitude environment, *LEF1* can protect the skin and eyes of Hetian sheep from ultraviolet radiation (Han et al., 2022). These studies suggest that *LEF1* is indispensable for sensory perception.

### OPRL1

Pain is a necessary protective response activated by high threshold stimulation. Animals that lack the ability to sense pain often suffer from infections and self-mutilation, resulting in shortened life spans (Woolf and Ma, 2007; Axelrod and Hilz, 2003). As a member of the opioid receptor gene family, *OPRL1* can directly inhibit neurons and then inhibit spinal cord pain (Al-Hasani and Bruchas, 2011). Another study confirmed that *OPRL1* might act as a potential biomarker that correlated with low back pain in patients (Zhao et al., 2020). Further studies are needed to confirm the function of *OPRL1* in pain.

### ATF6

Activating transcription factor 6 (*ATF6*) is a transcription factor belonging to the leucine zipper family. It has been confirmed that *ATF6* performed key functions in regulating the unfolded protein response (UPR) pathway, which is necessary for transducing stress signals to endoplasmic reticulum (Kohl et al., 2015; Hillary and FitzGerald, 2018). The mutation of *ATF6* impaired the development of fovea and cone photoreceptors in humans, which is a new cause of achromatopsia (Xu et al., 2015; Chan et al., 2016). These findings suggested that ATF6 performed key functions in visual development.

### MYO1A

The myosin superfamily is a versatile group of molecular motors involved in a number of cellular pathways (Hartman et al., 2012). As one member of class-1 myosins, *MYO1A* is a candidate gene associated with tolerance to heat stress in cattle (Cao et al., 2022). Besides, it has been reported that mutations in *MYO1A* can cause non-syndromic sensorineural hearing loss (F. Talebi et al., 2018). Moreover, comparative genomic analysis between Xiang pigs and other breeds found *MYO1A* was a selected candidate gene involved in environmental adaptation, the study further speculated p.V299IA splice variant of *MYO1A* may regulate coat color or hearing in Xiang pigs (Wang et al., 2022). Further experiments in vivo and vitro are needed to confirm this speculation.

### RDH5

Retinol is a common fat-soluble vitamin essential for animal visual function, embryonic development, immune regulation, reproduction, cell growth, and differentiation, etc. (Saari et al., 2004; Yadu and Kumar, 2019; Clagett-Dame and Knutson, 2011; Huang et al., 2018; Polcz and Barbul, 2019; Ayuso et al., 2015; Yang et al., 2018). However, retinol must produce active retinoic acid to perform its function (Kedishvili et al., 2013). Retinol dehydrogenase, also known as RDH, is mainly distributed in the endoplasmic reticulum of the photoreceptor inner segments. It can catalyze the conversion between all-trans-retinol and all-trans-retinol or the reduction of retinaldehyde using nicotinamide adenine dinucleotide (NAD) or nicotinamide adenine dinucleotide phosphate (NADP) as cofactors and participate in the first step of the visual cycle. Besides, RDH has a detoxification effect on the products of lipid peroxidation in photoreceptors. Once abnormal, it will lead to progressive photoreceptor degeneration, which affects the photoreceptor cells of rods and cones and causes blindness. Notably, scholars have reviewed the main reactions in the rod visual cycle, and RDH plays an important role in this process (Sahu and Maeda, 2016). These findings suggested that the *RDH5* gene encoding retinol dehydrogenase performed key roles in vision development.

Missense mutation may affect the structure and function of enzyme protein. The current study found that T722C mutation in *RDH5* only existed in the wild sheep population and changed amino acid residues at the 241 ${}_{\mathrm{th}}$ in its protein, which is located on the 241 ${}_{\mathrm{th}}$ loop region and adjacent to the α 8 and α 9 helix. Besides this, a comparison of 3D protein structure between wild and mutated revealed that mutation shortened the α 8 helix and caused the two alpha helixes ( α 8 and α 9) to keep away from each other. Meanwhile, the variation of amino acid also changed the hydropathicity, which can affect the stability of protein. Scholars found that the mutation of the hydrophilic residue from xylanase increased protein stability (Gallardo et al., 2010). Our study also found that variation of amino acid residues at the 241Gallardo in *RDH5* protein reduced the hydropathicity and weakened the enzymatic activity of retinol dehydrogenase.

As a key encoded enzyme by the *RDH5* gene, 11-*cis*-retinol dehydrogenase is found abundantly expressed in the retinal pigment epithelial cells (Liu et al., 2015). Mutations in *RDH5* result in a decrease of the enzyme activity that leads to serious retinopathy. Recent studies investigated the clinical characteristics of a night blindness named familial adenomatous polyposis (FAP) in a Japanese cohort, which was influenced by the *RDH5* gene. Eight variants of *RDH5* were identified in these patients. Meanwhile, the high incidence of macular disease and decreased pyramidal response were found in elderly patients (Katagiri et al., 2020). Therefore, this evidence suggested that the T722C mutation of *RDH5* may impair the vision cycle in the wild sheep population, we infer that the *C* allele disappeared in the sheep domestication process.

## 5 Conclusions

According to the selection sweeps caused by artificial and natural selection, this study revealed that the genes related to sensory perception pathways had directional evolution in the autosomal region, such as *OPRL1, LEF1, TAS1R3, ATF6, VSX2, MYO1A, RDH5*, and some novel genes. The T722C polymorphism of the *RDH5* gene is a new positive selected site only existing in the Asiatic mouflon sheep population when compared with Hu sheep, which can be inferred to influence the vision cycle via affecting the activity of retinol dehydrogenase.

## Supplement

10.5194/aab-66-81-2023-supplement The supplement related to this article is available online at: https://doi.org/10.5194/aab-66-81-2023-supplement.

## Data Availability

The whole genome resequencing datasets of 49 Hu sheep supporting the conclusions of this article have submitted in the NCBI Sequence Read Archive under BioProject ID PRJNA675390 and PRJNA691115. The downloaded resequencing data from NCBI database supporting the conclusions of this article are available in the NCBI Sequence Read Archive under accession number SRP066883 (Pan et al., 2018) and PRJNA624020 (Li et al., 2020).
