# Supplementary material for: Selection signature analysis reveals RDH5 performed key function in vision during sheep domestication process
Source: Arch Anim Breed. 2023 Feb 23;66(1):81–91. doi: 10.5194/aab-66-81-2023 (PMC10294028; doi:10.5194/aab-66-81-2023)
Supplement: The supplement related to this article is available online at: https://doi.org/10.5194/aab-66-81-2023-supplement. [file aab-66-81-supplement.zip › aab-66-81-2023-supplement-title-page.pdf]

Supplement of Arch. Anim. Breed., 66, 81–91, 2023
https://doi.org/10.5194/aab-66-81-2023-supplement
© Author(s) 2023. CC BY 4.0 License.

Open Access
Archives Animal Breeding

Supplement of

Selection signature analysis reveals *RDH5* performed key function in vision during sheep domestication process

Ruixue Hu et al.

Correspondence to: Guiqiong Liu (liuguiqiong@mail.hzau.edu.cn)

- aab-66-81-2023-supplement-title-page.pdf
- Supplementary material-R2.xlsx

The copyright of individual parts of the supplement might differ from the article licence.
